# Enhancing pili assembly and biofilm formation in *Acinetobacter baumannii* ATCC19606 using non-native acyl-homoserine lactones

**DOI:** 10.1186/s12866-015-0397-5

**Published:** 2015-03-07

**Authors:** Li-mei Luo, Li-juan Wu, Yu-ling Xiao, Dan Zhao, Zhi-xing Chen, Mei Kang, Qi Zhang, Yi Xie

**Affiliations:** Department of Laboratory Medicine, West China Hospital of Sichuan University, Chengdu, 610041 China

**Keywords:** *A. baumannii*, Bacteria, Quorum sensing, Type IV pili, Biofilm, Bacterial adhesion

## Abstract

**Background:**

Quorum Sensing (QS) systems influence biofilm formation, an important virulence factor related to the bacterial survival and antibiotic resistance. In *Acinetobacter baumannii*, biofilm formation depends on pili biosynthesis, structures assembled via the csuA/BABCDE chaperone-usher secretion system. QS signaling molecules are hypothesized to affect pili formation; however, the mechanism behind this remains unclear. This study aimed to demonstrate the possible role of QS signaling molecules in regulating pili formation and mediating the ability to form biofilms on abiotic surfaces.

**Results:**

Real-time quantitative PCR analysis showed the expression of the csuA/BABCDE genes distinctly increased when co-cultured with C6-HSL (*P* < 0.05). Under the same experimental conditions, expression of *BfmS* and *BfmR* was significantly higher than the control strain (*P* < 0.05). A subsurface twitching assay showed a switch from a small to a large and structured clone that may result from enhanced twitching motility (*P* < 0.05). Transmission electron microscopy analysis of cells lifted from a MH broth co-cultured with C6-HSL showed more abundant pili-like structures than the control strain. We then tested the idea that the addition of a QS signal, and therefore induction of chaperone-usher secretion system genes, provides a greater benefit at higher biofilm densities. An assay for the total fluorescence intensity of the biofilm using Confocal Laser Scanning Microscopy revealed an obvious increase.

**Conclusion:**

Our study demonstrated that, increased transcription of the *BfmS* and *BfmR* genes, QS signaling molecules enhance the expression of the chaperone-usher secretion system, and this expression is required for twitching motility in *A. baumannii*. The concomitant pili expression and strain twitching allowed *A. baumannii* to attach easily to abiotic surfaces and form biofilms at an earlier timepoint.

## Background

*Acinetobacter baumannii* is an important Gram-negative nosocomial pathogen often associated with severe nosocomial infections, including ventilator-associated pneumonia, urinary tract infections, bacteremia and septicemia, especially in patients hospitalized in intensive care units [1,2]. *A. baumannii* is highly resistant to several antimicrobial agents, conferred mainly by intrinsic expression of cephalosporinase and efflux pumps, and by formation of biofilms [3]. The biofilms of *A. baumannii* lead to a reduction in the accumulation of antibiotics in the biofilm polymeric matrix [4]. Biofilms are also associated with survival properties, virulence expression and bacterial communication [5,6]. Recent studies indicate biofilm development is related to quorum sensing. Quorum sensing is an important global regulatory system in bacteria that provides a mechanism to coordinate the behavior of individual bacteria in a population [7]. Biofilms provide a tertiary structure for bacterial communication mediated by quorum sensing pathways. A number of signaling molecules with the ability to modulate quorum sensing-dependen enzymes are known as regulators for biofilm formation [8,9]. In Gram-negative species, acyl-homoserine lactones (AHLs) are mainly employed as autoinducers used by bacteria to control biofilm formation and maintenance [10,11]. Along with many kinds of AHLs, the production of C6-HSL was previously found in *A. baumannii* clinical isolates [12], and most Acinetobacter strains showed very weak degradation activity against C6-HSL [13].

Pili of *A. baumannii* are encoded by the csuA/BABCDE chaperone-usher assembly system, which is controlled by a two-component regulatory system encoded by *BfmS* and *BfmR*. It was previously shown that *BfmR* is essential for stabilization of *csu* operon expression and the expression of *csuC* and *csuE* genes is involved in the initial surface attachment during biofilm formation [5,14]. These data suggest *A. baumannii* pili are a key factor in biofilm formation. Although quorum sensing and bacterial pili have been implicated in *A. baumannii* biofilm formation, there is very little known about the mechanism surrounding these signal molecules, *csuA/BABCDE*-mediated pili and biofilms in *A. baumannii*. In this study, an analysis of the processes of pili production and surface attachment of *A. baumannii* ATCC19606 was initiated, including the associated gene expression of csuA/BABCDE chaperone-usher complex and their regulating genes (*BfmS*/*R*). In addition, we present evidence for a possible role of quorum sensing signaling molecules in the formation of biofilms on abiotic surfaces.

## Results and discussion

### Impact of C6-HSL on chaperone-usher complex expression

**Figure 1:**
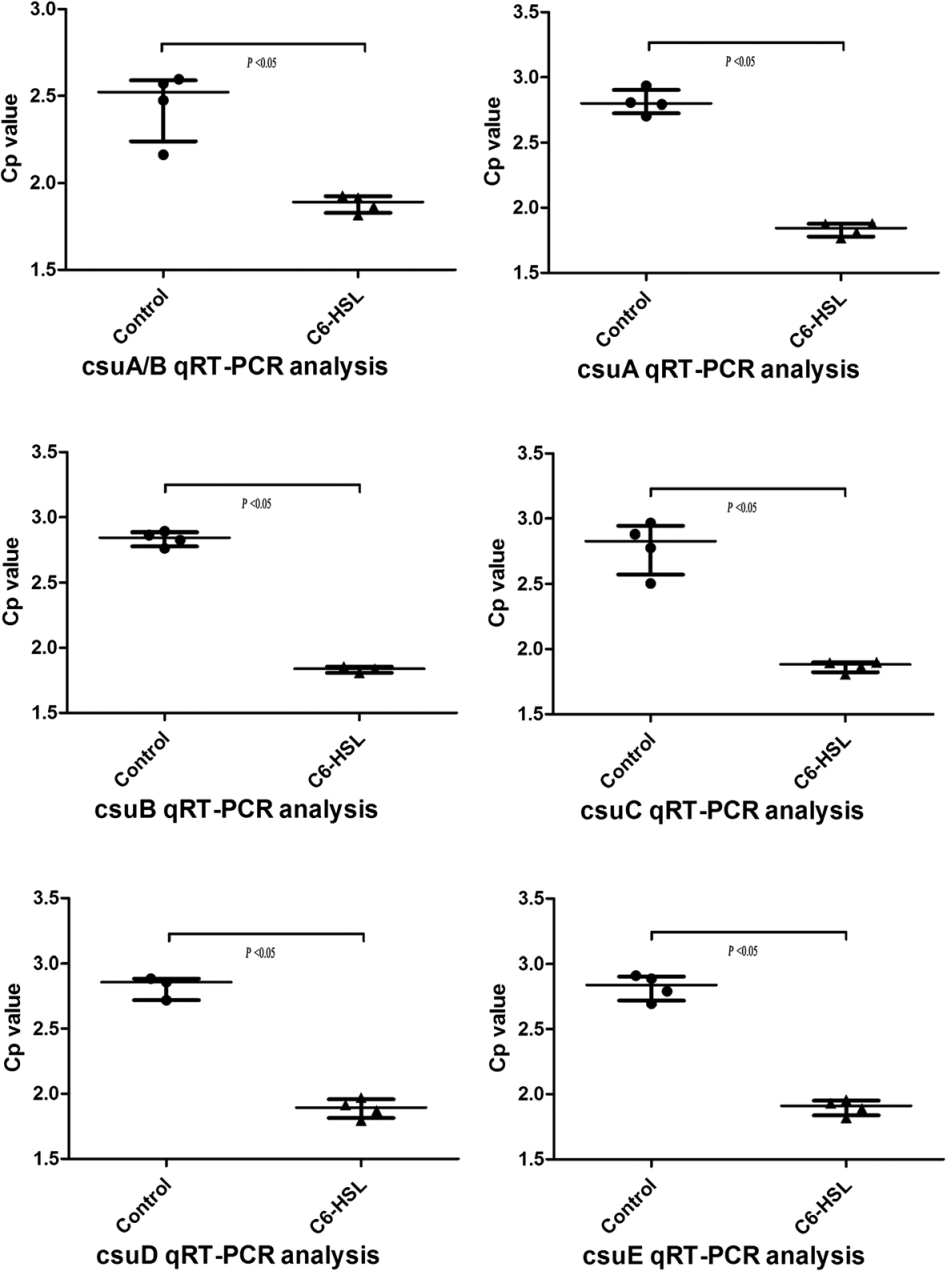
**Transcript levels of genes within the** ***csu*** **operon.** Quantitative RT-PCR assays of ATCC19606 cells grown in LB broth without AHLs (control) or with the addition of 100 μmol/L AHLs (C6-HSL). Transcription of each gene of the chaperone-usher complex were increased >1.5-fold.

**Figure 2:**
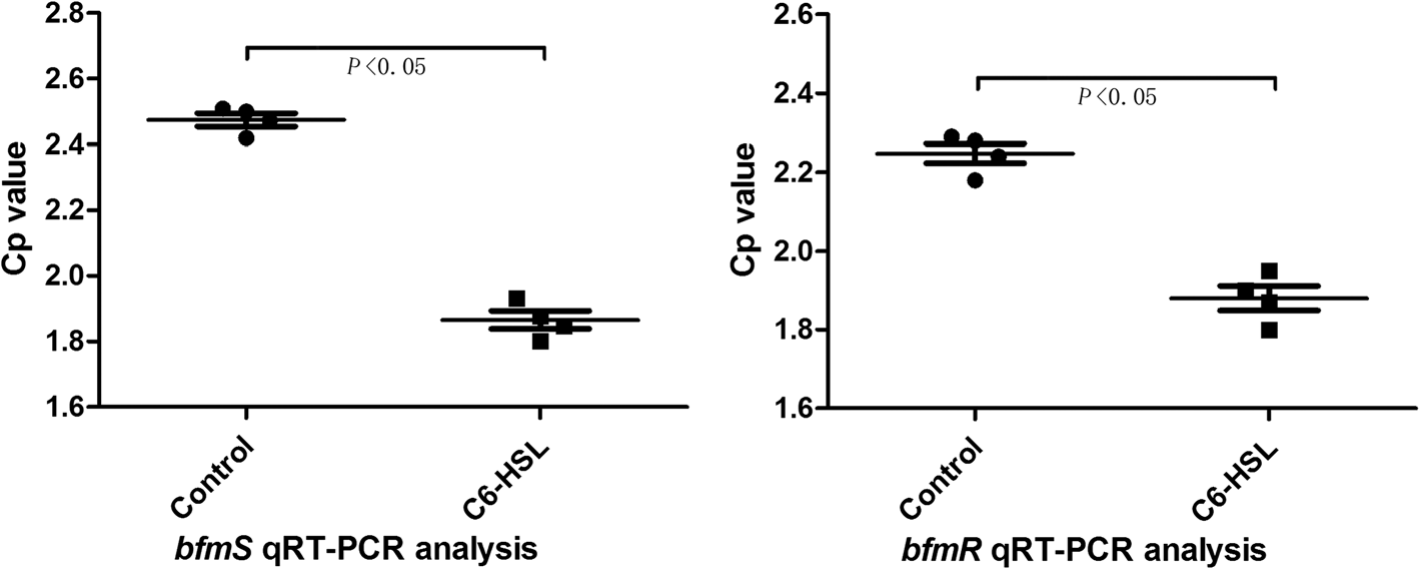
**Transcript levels of the csuA/BABCDE chaperone-usher complex regulating genes** ***BfmS/R.*** Quantitative RT-PCR assays of ATCC19606 cells grown in LB broth without AHLs (control) or with the addition of 100 μmol/L AHLs (C6-HSL). Both genes were increased approx 1.33-fold.

The capacity of *A. baumannii* to form biofilms is a decisive advantage for its survival in the hospital environmental. Recent studies have linked biofilm development with quorum-sensing pathways and bacterial factors, such as *A. baumannii* pili [15,16]. It is known that disruption of the *csuC* and *csuE* ORFs, which belong to the csuA/BABCDE bacterial pili structure gene cluster, results in non-piliated cells and abolishes cell attachment [14]. However, the exact mechanism of how QS pathways and *csu* influence biofilm formation is unclear. To directly examine all the genetic components of the csuA/BABCDE, and their regulators, the *BfmS-BfmR* regulating system that includes response factor (*BfmR*) and sensor kinase (*BfmS*), we provide data on the comprehensive expression of the pili structure gene cluster and the impact of C6-HSL on this chaperone-usher secretion system. Our results showed expression of bacterial pili structure genes, including *csuA/B*, *csuA*, *csuB*, *csuC*, *csuD* and *csuE*, significantly increased after addition of 100 μmol/L C6-HSL, and the transcript levels of the csuA/BABCDE chaperone-usher complex were increased >1.5-fold over the control group (*P* < 0.05, Figure 1). Furthermore, at the same experimental conditions, expression of chaperone-usher regulators (*BfmS* and *BfmR*) were higher than those of the control strain, and the regulators were increased approx 1.33-fold (*P* < 0.05, Figure 2).

### Subsurface twitching motility and transmission electron microscopy

**Figure 3:**
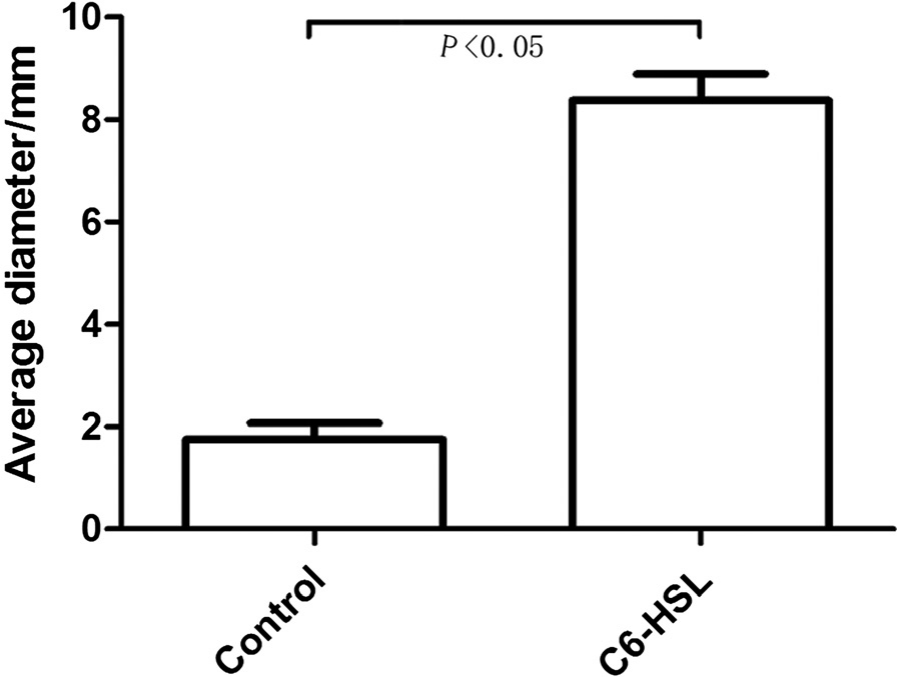
**Impact of C6-HSL on twitching motility.** Four individual colonies grown on separate plates incubated at 37°C for 24 h. The twitching zones were stained and their diameters measured at least three times. Results shown represent the means ± standard deviations.

**Figure 4:**
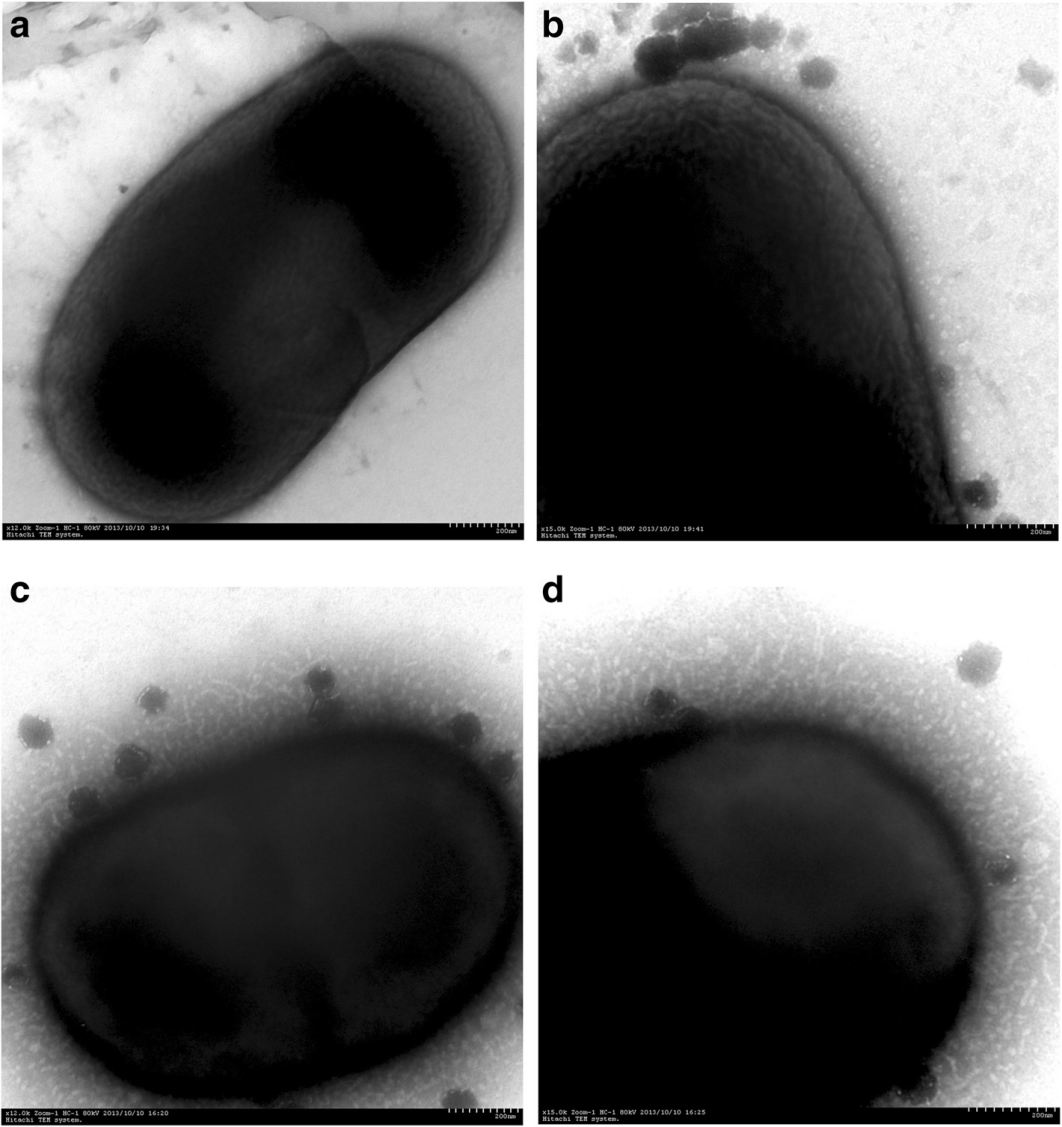
**TEM images of an** ***A. baumannii*** **bacterium grown in solution with or without C6-HSL.** TEM images were captured at magnification of ×12,000 (left column) and at ×15,000 (right column). **(a)** 12, 000-power magnification and **(b)** 15,000-power magnification of bacterial cell grown on glass slips incubated in MH broth without shaking over night at 37°C. **(c)** 12,000-power magnification and **(d)** 15,000-power magnification of bacteria cell growth in MH with 100 μmol/L C6-HSL forms obvious pili-like structures.

Despite the lack of flagella, *A. baumannii* can spread rapidly over surfaces, probably due to twitching motility [17]. Twitching is a form of surface motility mediated by type IV pili [18]. In *Pseudomonas aeruginosa*, twitching has been implicated in biofilm development [19] and a correlation has been found between twitching motility activity and biofilm production [20]. To determine whether C6-HSL affects bacterial twitching motility, we performed a subsurface twitching assay at the agar/glass interface comparing the diameter of twitching motility zones between the control group and *A. baumannii* treated with 100 μmol/L of C6-HSL. The results showed that *A. baumannii* co-cultured with 100 μmol/L C6-HSL had markedly increased movement from 1.75 to 8.38 mm in 24 hours (*P* < 0.05, Figure 3), which may result from enhanced twitching motility. Transmission Electron Microscopy (TEM) was used to confirm that pili formation in *A. baumannii* cells was stimulated by C6-HSL. The TEM showed there were abundant pili-like structures around the bacteria treated with C6-HSL, while structures of pili were not observed on the top of the control bacterial cells (Figure 4).

### Confocal laser scanning microscopy

**Figure 5:**
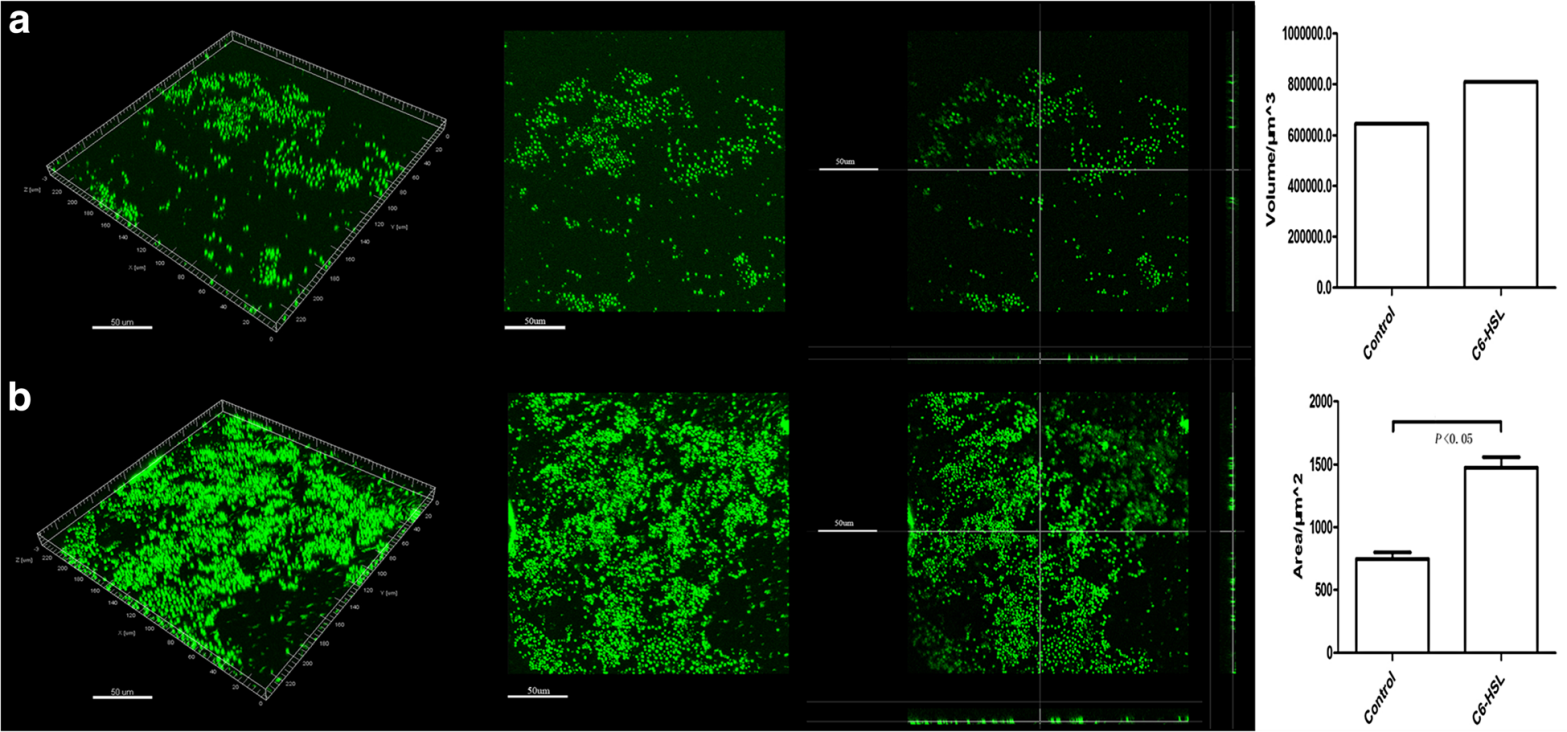
**Impact of C6-HSL on** ***A. baumannii*** **ATCC19606 biofilm formation.** The three-dimensional reconstruction of biofilms **(a)** in MH medium and **(b)** with 100 μmol/L C6-HSL added into MH medium were reconstructed after 4 days cultured without shaking at 37°C. The total fluorescence intensity, including **(c)** fluorescence volume and **(d)** fluorescence area was analyzed, and the results were averaged from three randomly selected positions of each sample.

If the increase in expression of bacterial pili seen in *A. baumannii* in response to C6-HSL is responsible for enhanced twitching motility, maintaining this quorum sensing stimulation in pili expression should obviously increase the capacity of the bacteria to form biofilms. With 100 μmol/L C6-HSL conditions, *A. baumannii* ATCC19606 was shown to form mature biofilms faster than the control group grown in MH medium, which yielded undeveloped biofilms. The results of the confocal laser scanning microscopy (CLSM) show the total fluorescence intensity of biofilms significantly increased in the C6-HSL group and the pili assembling from the surface of the cell was more abundant after C6-HSL stimulation (Figure 5).

A recent study [21] reported that a strain of *A. baumannii* with a *BfmS* knockout displayed a reduction in biofilm formation, loss of adherence to eukaryotic cells and greater sensitivity to serum killing. Our results demonstrated the expression of *BfmS* and *BfmR* regulated their target genes, the family of csuA/BABCDE chaperone-usher secretion system genes, to produce and assemble bacterial pili. Taken together, the result that the csuA/BABCDE chaperone-usher secretion system was essential to bacterial loci encoding secretion and surface motility (required in the early steps of biofilm formation) combined with our twitching assay results led us to conclude C6-HSL may promote *A. baumannii* pilus biosynthesis and assembly, as well as strain twitching ability, thereby ensuing formation of biofilms. However, QS signaling molecules are chemically diverse and many bacteria possess more than one AHL synthase [22]. In *A. baumannii,* many other QS signaling molecules have been verified, such as 3-oxo-C12-HSL, 3-hydroxy-C12-HSL and C8-HSL [12,23]. It is important to keep in mind that we focused only on the impact of C6-HSL on *A. baumannii*, which limited our study. In the future, it would be important to test other AHLs commonly produced by *A. baumannii* and other bacteria.

## Conclusion

In summary, we provided data demonstrating how, increased expression of *BfmS* and *BfmR*, the QS signaling molecule C6-HSL enhanced expression of the chaperone-usher secretion system, and that bacterial pili are required for twitching motility in *A. baumannii*. Furthermore, the concomitant pili expression and strain twitching allowed *A. baumannii* to easily attach to abiotic surfaces and form biofilms at an earlier timepoint. QS signaling molecules are required for cell attachment to solid surfaces and the development of biofilms. Our study describes the biofilm formation of *A. baumannii* in response to a QS signaling molecule, a finding that provides a comprehensive insight into the role of bacterial pili, which play a key role in bacterial biofilm development.

## Methods

### Strains and culture conditions

*A. baumannii* ATCC19606 was routinely growth in Luria–Bertani (LB) broth. Strains were grown at 37°C with shaking (220 rpm). *N*-Hexanoyl-L-homoserine lactone (C6-HSL, C_10_H_17_NO_3_, 100 μmol/L), purchased from Cayman (Cayman Chemical, Ann Arbor, MI, USA), was added to LB broth for co-culture with *A. baumannii*. Cells were harvested 12 h after inoculation, resuspended in Trizol reagent (TaKaRa, Japan) and stored at −80°C until use.

### RNA isolation and quantitative RT-PCR

Following the manufacturer’s recommendations, RNA was extracted using the MiniBEST Universal RNA Extraction Kit (Takara Bio, Shiga, Japan) and then the RNA concentration was adjusted before reverse transcription to avoid differences in gene expression due to different initial amounts of template. After RNA reverse transcription using One Step PrimeScript™ RT-PCR Kit (Takara Bio, Shiga, Japan), Quantitive Real-Time PCR (qRT-PCR) was performed by LightCycler®480 System ((Roche Diagnostic Systems, Mannheim, Germany). The primers used in this study are listed in Table 1. All qRT-PCR assays were repeated at least three times.

**Table 1:** **Oligonucleotides used for qRT-PCR in this study**

**Locus tag**	**Forward primer (5′-3′)**	**Reverse primer (5′-3′)**
16s DNA*	GTAATACAGAGGGTGCGAGCGTT	TCTAGCTGACCAGTATCGAATGCA
***csuA/B***	CAGCAGCAACAGGTGGCAATA	AAGGTTTGTACGTGCAGCATCA
***csuA***	TATTGCCTTCTTGTTCTG	CAGTTGAAATACCAGCAC
***csuB***	TATGCAGCAGATCCTCAG	TAAACTTTCCGTACAACG
***csuC***	TGGTCAGAAGTTTGCGCGTC	ACCAGAACTGTCCACACCATAAATT
***csuD***	CCGGTTCCCTAATTTATATGGCA	TAAGGCGTCACCGATGGCA
***csuE***	GCTTGGCTTTAGCAAACATGACC	ATTGCCATCAGGCCCGCTA
***BfmS***	ACCGCCCGTAATCCGAAC	TGAACTTATTCCACCGCCTTTA
***BfmR***	GTTTAACCGTTTGTCGTG	GTGGTTGAACTGGTTTCG

*Oligonucluotides used as references.

### Subsurface twitching assay

Surface-associated twitching motility was measured by a method described previously [24]. Briefly, 100 μmol/L C6-HSL was added to Mueller-Hinton (MH) medium solidified with 1% agar. The *A. baumannii* colony was stab-inoculated through the agar to the underlying Petri dish and covered by a glass cover slip on the inoculation site. After incubation at 37°C for 24 h, the cover slips were carefully lifted up, washed with phosphate-buffered saline (PBS) and stained with 0.1% crystal violet (wt/vol) for 1 minute. To remove excess crystal violet, each cover slip was gently washed with PBS and allowed to dry. The visualized diameter of twitching motility zones in C6-HSL concentration and untreated MH were measured at least three times.

### Transmission electron microscopy analysis

For TEM analysis, glass cover slips, lifted from MH culture medium, were immediately flooded with 2.5% glutaraldehyde and incubated at 4°C at least 2 h. Then, slips were rinsed with distilled water and dehydrated with increasing concentrations of ethanol ranging from 25 to 100% before being CO_2_ critical point dried. Samples were negative-stained with 1% phosphotungstic acid and visualized with HC-1 Hitachi TEM SYSTEM (Hitachi, Japan).

### Biofilm formation and CLSM assay

A static biofilm formation assay was performed as described previously [25]. Briefly, an overnight culture of *A. baumannii* ATCC19606 was subsequently diluted 100-fold in fresh MH broth in polystyrene microtiter plates (Corning, New York, NY, USA) with a sterilized glass cover slip in each well. C6-HSL (final concentration 100 μmol/L) was added, and the plates were incubated at 37°C for 4 days. After biofilms occupied the surface of slips, planktonic cells of *A. baumannii* were washed by PBS three times. SYTO® 9 Nucleic Acid Stain Acce (Invitrogen Corporation, Carlsbad, CA, USA) was used to label the biofilms developed by bacterial cells. After 15-min incubation with SYTO® 9, the cover slips were sealed for CLSM (DM IRE 2, Leica Microsystems, Germany). Under the particular wavelengths of 488 nm (absorption maxima) and 498 nm (emission maxima), each sample was scanned in at least three randomly selected positions, and the three-dimensional reconstruction of the biofilms was performed.

### Data analysis

T-test of independent sampler was performed to compare two groups by software SPSS 16.0 for Windows, and the *p* < 0 .05 was considered statistically significant.

## Abbreviations

QS: Quroum sensing
*A.baumannii*: *Acinetobacter baumannii*
CLSM: Confocal laser scanning microscopy
TEM: Transmission electron microscopy
C6-HSL: *N*-Hexanoyl-L-homoserine lactone
3-oxo-C12-HSL: *N*-(3-oxo-dodecanoyl)-L-homoserine lactone
3-hydroxy-C12-HSL: *N*-(3-hydroxydodecanoyl)homoserine lactone
C8-HSL: *N*-octanoyl-L-homoserine lactone

## References

[1] Dijkshoorn L, Nemec A, Seifert H (2007). An increasing threat in hospitals: multidrug-resistant *Acinetobacter baumannii*. Nat Rev Microbiol.

[2] Joly-Guillou ML (2005). Clinical impact and pathogenicity of Acinetobacter. Clin Microbiol Infect.

[3] Peleg AY, Seifert H, Paterson DL (2008). *Acinetobacter baumannii*: emergence of a successful pathogen. Clin Microbiol Rev.

[4] Espinal P, Marti S, Vila J (2012). Effect of biofilm formation on the survival of *Acinetobacter baumannii* on dry surfaces. J Hosp Infect.

[5] Gaddy JA, Actis LA (2009). Regulation of *Acinetobacter baumannii* biofilm formation. Future Microbiol.

[6] Longo F, Vuotto C, Donelli G (2014). Biofilm formation in *Acinetobacter baumannii*. New Microbiol.

[7] Camilli A, Bassler BL (2006). Bacterial small-molecule signaling pathways. Science.

[8] Worthington RJ, Richards JJ, Melander C (2012). Small molecule control of bacterial biofilms. Org Biomol Chem.

[9] Stacy DM, Welsh MA, Rather PN, Blackwell HE (2012). Attenuation of quorum sensing in the pathogen *Acinetobacter baumannii* using non-native *N*-Acyl homoserine lactones. ACS Chem Biol.

[10] Garg N, Manchanda G, Kumar A (2014). Bacterial quorum sensing: circuits and applications. Antonie Van Leeuwenhoek.

[11] Fuqua C, Parsek MR, Greenberg EP (2001). Regulation of gene expression by cell-to-cell communication: acyl-homoserine lactone quorum sensing. Annu Rev Genet.

[12] Chan KG, Cheng HJ, Chen JW, Yin WF, Ngeow YF (2014). Tandem mass spectrometry detection of quorum sensing activity in multidrug resistant clinical isolate *Acinetobacter baumannii*. Sci World J.

[13] Ochiai S, Morohoshi T, Kurabeishi A, Shinozaki M, Fujita H, Sawada I (2013). Production and degradation of *N*-acylhomoserine lactone quorum sensing signal molecules in bacteria isolated from activated sludge. Biosci Biotechnol Biochem.

[14] Tomaras AP, Dorsey CW, Edelmann RE, Actis LA (2003). Attachment to and biofilm formation on abiotic surfaces by *Acinetobacter baumannii*: involvement of a novel chaperone-usher pili assembly system. Microbiology.

[15] Solano C, Echeverz M, Lasa I (2014). Biofilm dispersion and quorum sensing. Curr Opin Microbiol.

[16] Chow JY, Yang Y, Tay SB, Chua KL, Yew WS (2014). Disruption of biofilm formation by the human pathogen *Acinetobacter baumannii* using engineered quorum-quenching lactonases. Antimicrob Agents Chemother.

[17] Wilharm G, Piesker J, Laue M, Skiebe E (2013). DNA uptake by the nosocomial pathogen *Acinetobacter baumannii* occurs during movement along wet surfaces. J Bacteriol.

[18] Burrows LL (2012). *Pseudomonas aeruginosa* twitching motility: type IV pili in action. Annu Rev Microbiol.

[19] Klausen M, Heydorn A, Ragas P, Lambertsen L, Aaes-Jorgensen A, Molin S (2003). Biofilm formation by *Pseudomonas aeruginosa* wild type, flagella and type IV pili mutants. Mol Microbiol.

[20] Wolska K, Kot B (2013). Twitching motility activity, biofilm formation, and genetic typing for clinical isolates of *Pseudomonas aeruginosa* by random amplified DNA PCR. Acta Microbiol Immunol Hung.

[21] Liou ML, Soo PC, Ling SR, Kuo HY, Tang CY, Chang KC (2013). The sensor kinase *BfmS* mediates virulence in *Acinetobacter baumannii*. J Microbiol Immunol Infect.

[22] Williams P (2007). Quorum sensing, communication and cross-kingdom signalling in the bacterial world. Microbiology.

[23] Chan KG, Atkinson S, Mathee K, Sam CK, Chhabra SR, Camara M (2011). Characterization of *N*-acylhomoserine lactone-degrading bacteria associated with the Zingiber officinale (ginger) rhizosphere: co-existence of quorum quenching and quorum sensing in Acinetobacter and Burkholderia. BMC Microbiol.

[24] Semmler AB, Whitchurch CB, Mattick JS (1999). A re-examination of twitching motility in *Pseudomonas aeruginosa*. Microbiology.

[25] Li M, Huang R, Zhou X, Zhang K, Zheng X, Gregory RL (2014). Effect of nicotine on dual-species biofilms of Streptococcus mutans and Streptococcus sanguinis. FEMS Microbiol Lett.

